# Supplementary material for: Consumption of Non-Nutritive Sweetener during Pregnancy and Weight Gain in Offspring: Evidence from Human Studies
Source: Nutrients. 2022 Dec 1;14(23):5098. doi: 10.3390/nu14235098 (PMC9739060; doi:10.3390/nu14235098)

Supplemental Table S1. Detailed search strategies used for the database search

a. for PubMed (via Medline)

Step	Detailed Search strategies
#1	"Sweetening Agents"[Mesh]
#2	Sweetener*[Title/Abstract]
#3	"Sugar Substitutes*"[Title/Abstract]
#4	"Non-Nutritive Sweeteners"[Mesh]
#5	OR/#1-4
#6	"Adolescent"[Mesh]
#7	"Infant"[Mesh]
#8	"Child"[Mesh]
#9	"Pediatrics"[Mesh]
#10	pediatric*[Title/Abstract]
#11	paediatric*[Title/Abstract]
#12	child*[Title/Abstract]
#13	infant*[Title/Abstract]
#14	adolescent*[Title/Abstract]
#15	neonat*[Title/Abstract]
#16	newborn*[Title/Abstract]
#17	teenager*[Title/Abstract]
#18	OR/#6-17
#19	"Pregnancy"[Mesh]
#20	Pregnan*[Title/Abstract]
#21	Gestat*[Title/Abstract]
#22	OR/#19-21
#23	#5 AND #18 AND #22

b. for EMBASE

Step	Detailed Search strategies
#1	'sweetening agent'/exp
#2	'nonnutritive sweetener'/exp
#3	sweetener*:ti,ab,kw
#4	'sugar substitutes*':ti,ab,kw
#5	OR/#1-4
#6	'adolescent'/ex
#7	'infant'/exp
#8	'child'/exp
#9	'pediatrics'/exp
#10	pediatric*:ti,ab,kw
#11	paediatric*:ti,ab,kw
#12	child*:ti,ab,kw

#13	infant*:ti,ab,kw
#14	adolescent*:ti,ab,kw
#15	neonat*:ti,ab,kw
#16	newborn*:ti,ab,kw
#17	teenager*:ti,ab,kw
#18	OR/#6-17
#19	'pregnancy'/exp
#20	pregnan*:ti,ab,kw
#21	gestat*:ti,ab,kw
#22	OR/#19-21
#23	#5 AND #18 AND #22

c. for Cochrane Library

**Step**	**Detailed Search strategies**
#1	MeSH descriptor: [Sweetening Agents] explode all trees
#2	('nonnutritive sweetener'):ti,ab,kw
#3	(sweetener*):ti,ab,kw
#4	('sugar substitutes*'):ti,ab,kw
#5	MeSH descriptor: [Adolescent] explode all trees
#6	MeSH descriptor: [Child] explode all trees
#7	MeSH descriptor: [Pediatrics] explode all trees
#8	MeSH descriptor: [Infant] explode all trees
#9	(pediatric*):ti,ab,kw
#10	(paediatric*):ti,ab,kw
#11	(child*):ti,ab,kw
#12	(infant*):ti,ab,kw
#13	(adolescent*):ti,ab,kw
#14	(neonat*):ti,ab,kw
#15	(newborn*):ti,ab,kw
#16	(teenager*):ti,ab,kw
#17	MeSH descriptor: [Pregnancy] explode all trees
#18	(pregnan*):ti,ab,kw
#19	(gestat*):ti,ab,kw
#20	(OR/#1-4) AND (OR/#5-16) AND (OR/#17-19)

Supplemental Figure S1. Scatter plots showing the different levels of NNS intake during pregnancy in relation to WMD in offspring at one year of age (the sizes of bubble plots reflecting the inverse variance of the WMD)

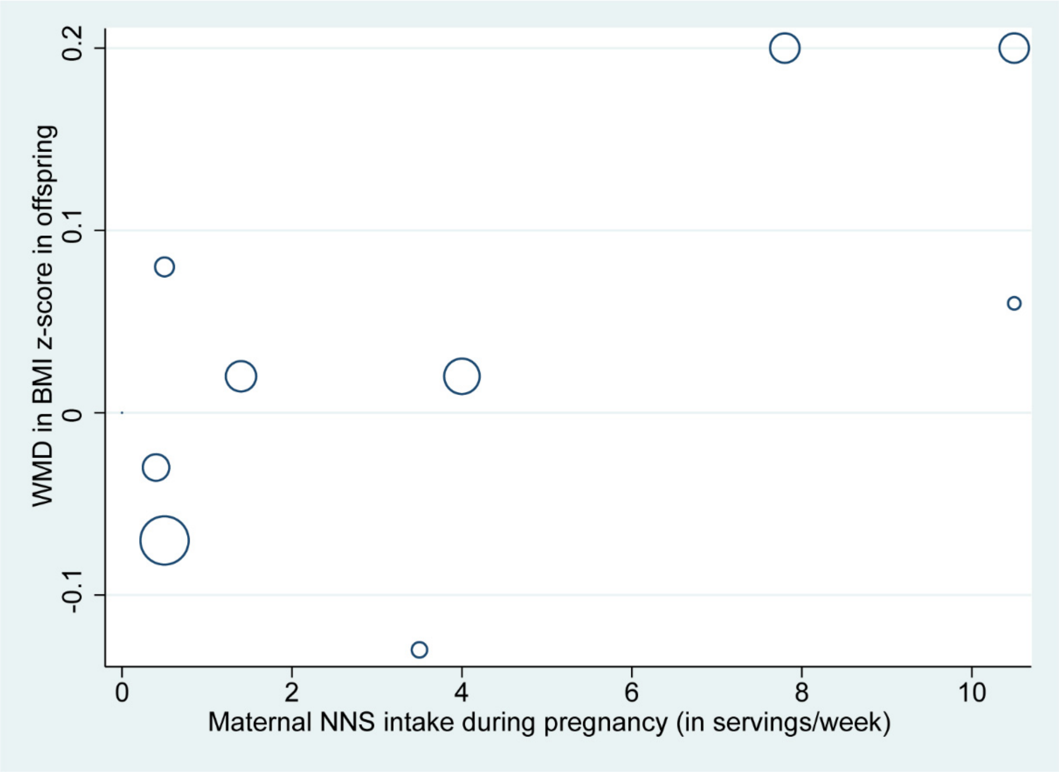

Supplemental Figure S2. : Scatter plots showing the different ages of offspring in relation to the WMD between offspring with and without maternal NNS intake, or between offspring with the highest and lowest level of maternal NNS intake (the sizes of bubble plots reflecting the inverse variance of the WMD)

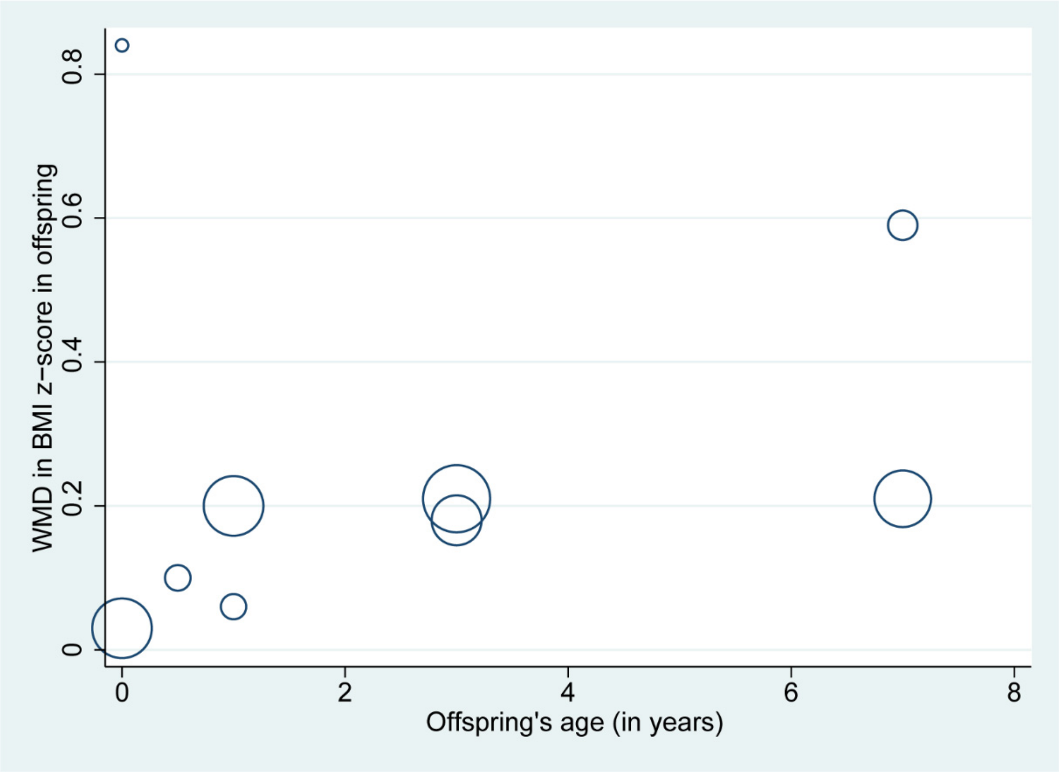

Supplemental Figure S3. Funnel plot for the relationship between NNS intake during pregnancy and WMD in offspring

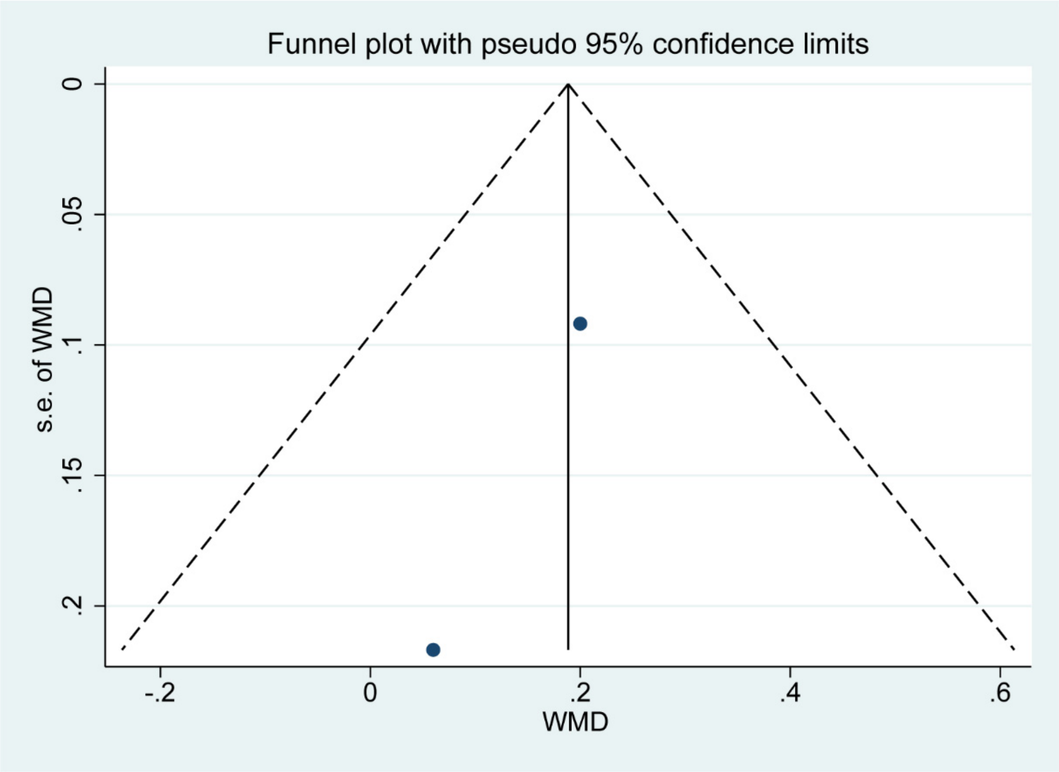

Supplement: Supplementary file 1 [file nutrients-14-05098-s001.zip › nutrients-2008875-supplementary.pdf]
